# Comparing the Efficacy of an Electronically Delivered Cognitive Behavioral Therapy Program to a Mental Health Check-In Program for Generalized Anxiety Disorder: Protocol for a Randomized Trial

**DOI:** 10.2196/48899

**Published:** 2023-09-20

**Authors:** Callum Stephenson, Anchan Kumar, Niloufar Malakouti, Niloofar Nikjoo, Jasleen Jagayat, Tessa Gizzarelli, Charmy Patel, Gilmar Gutierrez, Amirhossein Shirazi, Megan Yang, Mohsen Omrani, Nazanin Alavi

**Affiliations:** 1 Department of Psychiatry Faculty of Health Sciences Queen's University Kingston, ON Canada; 2 Centre for Neuroscience Studies Faculty of Health Sciences Queen's University Kingston, ON Canada; 3 OPTT Inc Toronto, ON Canada

**Keywords:** anxiety, cognitive behavioral therapy, eHealth, electronic care, generalized anxiety disorder, internet, mental health, psychotherapy, treatment, web-based

## Abstract

**Background:**

Generalized anxiety disorder (GAD) is a prevalent anxiety disorder, with cognitive behavioral therapy (CBT) being the gold standard treatment. However, it is inaccessible and costly to many, as the mental health industry is overwhelmed by the demand for treatment. This means effective, accessible, and time-saving strategies must be developed to combat these problems. Web-based interventions for mental health disorders are an innovative and promising way to address these barriers. While electronically delivered CBT (e-CBT) has already proved productive and scalable for treating anxiety, other less resource-intensive interventions can be innovated. Checking up on mental health face-to-face has been shown to provide similar benefits to patients with anxiety disorders previously, but more research is needed to evaluate the efficacy of web-based delivery of this intervention.

**Objective:**

This study will compare the efficacy of e-CBT and a web-based mental health check-in program to treat GAD. These programs will both be delivered through a secure, web-based care delivery platform.

**Methods:**

We will randomly allocate participants (N=100) who are 18 years or older with a confirmed diagnosis of GAD to either an e-CBT program or a mental health check-in program over 12 weeks to address their anxiety symptoms. Participants in the e-CBT arm will complete predesigned modules and homework assignments while receiving personalized feedback and asynchronous interaction with a therapist through the platform. Participants in the mental health check-in arm will be contacted weekly through the web-based platform’s written chat feature (messaging system). Therapists will ask the participants a series of predesigned questions that revolve around a different theme each week to prompt conversation. Using clinically validated questionnaires, the efficacy of the e-CBT arm will be compared to the mental health check-in arm. These questionnaires will be completed at baseline, week 6, and week 12.

**Results:**

The study received ethics approval in April 2021, and participant recruitment began in May 2021. Participant recruitment has been conducted through targeted advertisements and physician referrals. Complete data collection and analysis are expected to conclude by August 2023. Linear and binomial regression (continuous and categorical outcomes, respectively) will be conducted.

**Conclusions:**

To the research team’s knowledge, this will be the first study to date comparing the efficacy of e-CBT with a web-based mental health check-in program to treat GAD. The findings from this study can help progress the development of more scalable, accessible, and efficacious mental health treatments.

**Trial Registration:**

ClinicalTrials.gov NCT04754438; https://classic.clinicaltrials.gov/ct2/show/NCT04754438

**International Registered Report Identifier (IRRID):**

DERR1-10.2196/48899

## Introduction

### Overview

Generalized anxiety disorder (GAD) is a prevalent and debilitating mental health disorder. More than 450 million people are living with mental and behavioral disorders, with many being unable to receive adequate treatment [1-3]. Cognitive behavioral therapy (CBT) is considered one of the first-line treatments for GAD; however, it is not without its drawbacks [4-7]. CBT is often inaccessible to patients because it requires in-person time commitment during work hours, travel to appointments, high costs, and potential exposure to social stigma [8]. CBT is extremely resource-intensive, requiring a large time commitment from a trained mental health professional. With the prevalence of mental health disorders as high as they are, the demand for this first-line therapy is not being met by North American health care systems, leaving them overwhelmed and patients unable to receive treatment [8,9]. Innovative interventions that optimize resources without sacrificing the quality of care are required to meet this demand.

Fortunately, web-based interventions for mental health disorders are growing in prevalence and are a promising way to address the barriers associated with in-person treatment, such as accessibility and convenience [10-15]. The structured nature of CBT has allowed it to be effectively adapted into a web-based format (electronically delivered CBT; e-CBT) where patients can access standard concepts remotely and in their own time. While e-CBT has already proven productive and scalable for treating anxiety disorders [10,16-19], developing less-intensive interventions that still benefit patients could allow for even more scalability. Not all patients may require the full structure of e-CBT and could still benefit from a less-intensive option, such as structured mental health conversations with a care provider. Checking in on individual mental health has previously shown benefits to patients with anxiety disorders, but much of this research has investigated in-person approaches and synchronous web-based deliveries [20-22]. Transferring this to a web-based asynchronous messaging format could allow for increased privacy, accessibility, temporal flexibility, and decreased cost and geographical barriers. Understanding how the delivery of e-CBT compares to a web-based delivery of mental health check-in conversations with a care provider can provide insight into the application of this as a viable intervention in the future.

### Objectives

The primary objective will be to compare the efficacy of an e-CBT program to a web-based mental health check-in program. Additionally, the individual effects of each treatment will be evaluated using clinically validated symptomology questionnaires.

## Methods

### Study Design

This study will use an open-label randomized controlled trial design to investigate the effects of e-CBT versus a web-based check-in program for GAD. Participants will be randomly assigned, in a ratio of 1:1, to either a 12-week e-CBT program tailored to GAD or a 12-week mental health check-in program to address their symptoms. In addition to these interventions, participants will continue with treatment as usual (ie, lifestyle, family physician appointments, and medications).

### Participants

Participants (total=100; e-CBT=50; and check-in=50) will be recruited in Kingston, Ontario, from outpatient psychiatry clinics at both Kingston Health Sciences Centre sites (Hotel Dieu Hospital and Kingston General Hospital). Additional recruitment from Providence Care Hospital, family doctors, physicians, clinicians, and self-referrals will occur. Once informed consent is obtained, participants will be evaluated by a psychiatrist on the research team through secure video appointments to make or confirm a diagnosis of GAD using the Diagnostic and Statistical Manual of Mental Disorders, 5th edition (DSM-5) [23].

Inclusion criteria include being aged 18 years or older at the start of the study, having a diagnosis of GAD according to the DSM-5 criteria by an attending psychiatrist on the research team, having the competence to consent to participate, having the ability to speak and read English, and having consistent and reliable access to the internet. Exclusion criteria include active psychosis, acute mania, severe alcohol or substance use disorders, and active suicidal or homicidal ideation. To avoid any confounding effect on the efficacy of this e-CBT program, participants currently receiving or having received CBT in the past year will also be excluded from this study to avoid confounding effects of treatment. If eligible for the study, participants will be randomly assigned to either the e-CBT program (n=50) or the mental health check-in program (n=50). Following the completion of the study, participants who were randomly assigned to the mental health check-in arm will be offered the e-CBT program if they are interested. During the informed consent process, it will be explained to all participants that this program is not a crisis resource and that they will not always have access to their therapist. In the case of an emergency, participants will be directed to the proper resources (ie, emergency department and crisis lines), and this event will be reported to the principal investigator.

### Procedures

#### CBT Arm

The e-CBT modules are designed to instill balanced and constructive coping strategies in participants. During the e-CBT program, the focus will be on essential thinking and behavioral skills to assist participants in becoming more engaged in day-to-day activities (Table 1). Additional focus will be placed on the connection between thoughts, behaviors, emotions, physical reactions, and the environment. All care providers are research assistants hired by the principal investigator. They all undergo training in psychotherapy delivery and additional training by the principal investigator on the research team, who is also a licensed therapist and an expert in web-based delivery, before interacting with participants. During this training, therapists complete feedback on practice homework, which is reviewed to ensure adequate quality of work. All care providers are supervised by a licensed therapist [10-12,16,24-26] who will review feedback before submission.

**Table 1:** Overview of the 12-week electronically delivered CBT^a^ program content.

Week	Session	Description
1	What is generalized anxiety disorder?	Provides expectations for the course and introduces anxiety and CBT.
2	The 5-part model	Introduces the concept of the 5-part model and how a situation, thoughts, feelings, physical reactions, and behaviors are connected and how they interact.
3	Strategies for stressful situations	Provides an overview of helpful strategies that can be used in stressful situations, including pleasurable activities and helpful breathing techniques.
4	Situation, thoughts, feelings, physical reactions, and behaviors	Provides a further detailed exploration of the 5-part model and how changes in one area can affect the other 4 parts.
5	The thought record	Highlights the first 3 columns of the thought record; a tool used to help understand the connection between feelings, behaviors, and thoughts. The first 3 columns include the situation, followed by the feelings and automatic thoughts associated with it.
6	Automatic thoughts	This delves into the role of automatic thoughts and how they influence feelings. The focus of this session is to understand how to identify automatic thoughts and specifically identify the most dominant idea, or “hot thought,” when presented with a stressful situation. Common thinking errors are also discussed in this session.
7	Activity scheduling	Provides a break from learning about the thought record and instead explains how to use an activity record; a tool designed to record and plan weekly activities. This session focuses on how tracking activities can inform mood changes and reinforce the scheduling of pleasurable activities.
8	Evidence	Focuses on the fourth and fifth columns of the thought record, which are designed to help gather the information that supports or does not support the identified hot thought.
9	Alternative and balanced thinking	Focuses on the final 2 columns of the thought record, which reflect on the evidence columns to help find an alternative or balanced view of the situation. The last column invites the viewer to rerate their feelings based on the completion of the thought record.
10	Experiments	Explain the importance of conducting experiments to start believing alternative or balanced thoughts from the thought record and initiating changes in ineffective thinking patterns.
11	Action plans	Centered around identifying a problem that needs to be solved and provides a framework for creating a plan for solving the problem.
12	Review	The final session is a review of the course and summarizes the main CBT concepts and tools that have been taught throughout the program.

^a^CBT: cognitive behavioral therapy.

The e-CBT care plan consists of 12 weekly sessions of approximately 30 slides and interactive content, delivered through the Online Psychotherapy Tool (OPTT), a secure, web-based, cloud-based mental health care delivery platform. The e-CBT module content will mirror in-person standard CBT content, including different weekly topics, general information, skill overviews, and homework. Participants will be instructed to go through the content and complete homework at the end of the session, which will help them practice the skills they learned during that session. Homework will be submitted directly through OPTT and reviewed by the care provider assigned to the participant, who will provide personalized feedback within 3 days of submission. Care providers will have access to predesigned session-specific feedback templates to use as a basic structure to write their feedback. Doing so reduces the time needed to respond to each patient; therefore, the number of patients each care provider can handle increases. On average, developing this feedback will take a care provider 15-20 minutes per patient. In addition to the weekly feedback, participants have the option to message their care provider through the platform throughout the week regarding any questions or concerns they may have.

#### Mental Health Check-In Arm

Participants in the mental health check-in arm will have weekly interactions with a care provider through the chat feature in OPTT. The care provider will reach out weekly using structured question prompts to initiate interaction with the patient (Table 2).

**Table 2:** Mental health check-in weekly procedures and question prompts.

Week	Topic	Question prompts
1	Mood	Tell me about yourself. How have you been feeling lately? How has the pandemic affected your mood? What differences have you noticed in your mood compared to the past?
2	Sleep	How have you been? Tell me about your week. I want to ask about your sleep. How many hours do you get? Do you feel rested? Do you think you need to make any changes to your sleep hygiene?
3	Activity	How was your week? How has your anxiety level been? What do you usually do to stay active during the day? Are there any indoor activities you can do at your place? Have you been using any online exercise programs?
4	Hobbies	What did you do in the past week? Do you have any hobbies? How long have you been doing them? Have you found any new activities that you enjoy recently?
5	Friendship	How was your week? How has the pandemic affected your relationship with your friends? Have you stayed in touch with your close friends during the pandemic? How often do you interact with your friends?
6	New events	How have you been? Have there been any new events in the past weeks? Are you planning to do anything new this week? Are you planning to do anything new this week? Are you expecting to hear any news from your family/friends?
7	Job and study	How have you been? Tell me about your week. How are you managing your day-to-day responsibilities (eg, job, school, personal, etc)?
8	Diet and food	How was your week? Let’s talk about your eating habits. How does your anxiety affect your appetite? Are you satisfied with your diet? How often do you cook? Do you prefer homemade food or take-out?
9	Books, movies, and television	How was your week? Has your anxiety affected your concentration? Do you like reading books? What is the last book you read? Are there any books you would recommend to your friends? What shows do you enjoy watching? What are some shows you would recommend to your friends?
10	Phone, apps, and games	Tell me about your week. How many hours a day do you usually spend on your phone? What is your favourite app? Do you play any games? What kinds of games do you like to play (eg, board games, cards, video games, etc)?
11	Habits	How have you been? How was your week? What do you think are some of your healthy habits? What are some habits you would like to change?
12	Accomplishments	How was your week? How did you find this program? Were the weekly check-ins helpful? Overall, was this program helpful for you? Why or why not? Are you interested in an online cognitive behavioural therapy program? Why or why not?

### Outcome Evaluation and Analysis

Participants in both arms will complete the following clinically validated questionnaires at baseline: State-Trait Anxiety Inventory (STAI) [27], Quality of Life Enjoyment and Satisfaction Questionnaire-Short Form (Q-LES-Q-SF) [28], Generalized Anxiety Disorder-7 item (GAD-7) [29], Depression Anxiety Stress Scale-42 item (DASS-42) [30], and a demographic questionnaire. All questionnaires will be completed through OPTT. The STAI, Q-LES-Q-SF, GAD-7, and DASS-42 will be completed again during week 6 and week 12. Using data from previous research incorporating this e-CBT program, a sample of 50 participants in each group was determined to be sufficient for an effect size of 0.5, a power of 0.8, and an α of .05 [31].

Before analysis, the data will be examined for missing, nonsensical, and outlying variables. Missing data will be removed on a per-protocol basis, with a statistical analysis significance level set at .05. Demographic information (ie, age, gender, and race) will be assessed between groups and program noncompleters using independent sample *t* tests. A 2×3 repeated measures ANOVA will be completed for each questionnaire score at the time points to investigate the differences between the 2 groups. An intention-to-treat analysis will be completed to assess whether the outcome of treatment impacts participants’ likelihood of not completing the program. Data will also be assessed with linear mixed-effects models with random effects as patient identification and fixed effects as treatment, time, and their interaction. SPSS (version 24; IBM Corp) will be used for all statistical analyses.

### Data Privacy

Participants will only be identifiable by an anonymous identification number, and all files are stored securely on a server with password encryption. Participant data will only be accessible by the care providers directly assigned to the participant, and anonymized data will be provided to the analysis team members. Participants can withdraw from the study at any point and request that their data be removed from the analysis.

OPTT is compliant with the Health Insurance Portability and Accountability Act, the Personal Information Protection and Electronic Documents Act, and Service Organization Control-2. Additionally, all servers and databases are hosted in the Amazon Web Service cloud infrastructure, which is managed by MedStack to assure all privacy and security regulations are met. OPTT does not collect any identifiable personal information or IP addresses for privacy purposes. OPTT only collects anonymized metadata to improve its service quality and provide advanced analytics to the clinician team. OPTT encrypts all data, and no employee has direct access to participant data. All encrypted backups will be kept in the S3 storage that is dedicated to Queen’s University.

### Ethic Approval

The study was approved (File 6032033) by the Queen’s University Health Science and Affiliated Teaching Hospitals Research Ethics Board in April 2021.

## Results

The recruitment of participants for this study began in May 2021. Participant recruitment has been conducted through social media advertisements, physical advertisements, and physician referrals. To date, 50 participants have been recruited. Data collection is expected to conclude by August 2023, and data analyses are expected to be completed by September 2024, with linear regression analysis (for continuous outcomes) and binomial regression analysis (for categorical outcomes) being conducted.

## Discussion

This study protocol outlines the methods to compare a 12-week e-CBT program and a 12-week check-in program for the management of GAD symptoms. Additionally, the individual effects of each treatment will be evaluated using clinically validated symptomology questionnaires.

GAD is a prevalent mental health disorder that demands resource-intensive interventions to properly address symptoms. CBT is currently one of the first-line treatments for GAD, but it tends to be inaccessible to patients and requires a high level of time commitment from mental health care professionals. Using the internet to deliver mental health treatments has become a promising solution in recent years, with e-CBT being scalable, accessible, and effective in treating GAD [16-18,32-34]. Now that e-CBT is an effective treatment option for patients with GAD, understanding ways to tailor the care to each patient, with varying intensity levels, is crucial for the future of scalability [11,12,16,24-26]. Checking in on patients’ mental health is another form of mental health care that can be less resource-intensive while still offering benefits to patients with GAD. Delivering this electronically and asynchronously would allow for more accessible access to treatment while also providing decreased time commitment, allowing more patients to be helped. There is less research on the impact of a check-in intervention over messaging services, identifying a knowledge gap. By directly comparing this check-in intervention to e-CBT, the efficacy and feasibility of implementing this as a treatment option in a web-based mental health clinic can be investigated. This comparison is original to previous literature, providing a direct comparison between e-CBT and mental health check-ins to investigate whether this is a viable resource to implement in a real-world setting. Most previous literature has investigated the comparison of e-CBT to in-person interventions, treatment groups, medications, or control groups, but none of the sort in this protocol, to the author’s knowledge.

The limitations of this protocol include the lack of a control group. However, the e-CBT program used in this protocol has been previously clinically validated [11], and the main objective of this study is to uncover the differences between these 2 treatment arms. Furthermore, this protocol does not currently include a long-term follow-up, which is something that could be explored in the future.

These findings can provide valuable insight into the effectiveness of a weekly mental health check-in from a care provider versus a validated 12-week e-CBT intervention. If the check-in intervention proves to be a viable and effective option, it could be used as a less resource-intensive option, used in conjunction with e-CBT, or as an option for patients presenting with less severe symptoms. By providing a framework for future research to use these forms of check-in interventions, more work can be done investigating the effects in conjunction with other treatments as well. Electronically delivered mental health interventions are an ever-expanding area with a focus needed on how to continue growing the reach to more patients without sacrificing accessibility and quality of care.

## Abbreviations

CBT: cognitive behavioral therapy
DASS-42: Depression and Anxiety Stress Scale-42 item
DSM-5: Diagnostic and Statistical Manual of Mental Disorders, 5th edition
e-CBT: electronically delivered cognitive behavioral therapy
GAD-7: Generalized Anxiety Disorder-7 item
OPTT: Online Psychotherapy Tool
Q-LES-Q-SF: Quality of Life Enjoyment and Satisfaction Questionnaire-Short Form
STAI: State-Trait Anxiety Inventory

## Data Availability

The data sets generated during and/or analyzed during this study will be made available from the corresponding author on reasonable request.
